# A Cystic-like Lesion of Uncertain Origin—A Discussion on Cemento-Osseous Dysplasia and Traumatic Bone Cysts

**DOI:** 10.3390/diagnostics15182312

**Published:** 2025-09-11

**Authors:** Kamil Nelke, Maciej Karpiński, Michał Scharoch, Maciej Janeczek, Agata Małyszek, Evagelos Kalfarentzos, Efthymios Mavrakos, Piotr Kuropka, Christos Perisanidis, Maciej Dobrzyński

**Affiliations:** 1 Maxillo-Facial Surgery Ward, EMC Hospital, Pilczycka 144, 54-144 Wrocław, Poland; 2 Maxillo-Facial Surgery Ward, Sokolowski Specialist Hospital, Walbrzych Sokolowski Str. 4, 58-309 Walbrzych, Poland; 3 Department of Biostructure and Animal Physiology, Wrocław University of Environmental and Life Sciences, Kożuchowska 1, 51-631 Wrocław, Poland; 4 Oral and Maxillo-Facial Surgery, University Clinic, Dental School, Evangelismos General Hospital, National and Kapodistrian University of Athens, 11527 Athens, Greeceefthymiosmav@med.uoa.gr (E.M.);; 5 Division of Histology and Embryology, Department of Biostructure and Animal Physiology, Wrocław University of Environmental and Life Sciences, Cypriana K. Norwida 25, 50-375 Wrocław, Poland; 6 Department of Pediatric Dentistry and Preclinical Dentistry, Wrocław Medical University, Krakowska 26, 50-425 Wrocław, Poland

**Keywords:** cemento-osseous dysplasia, traumatic bone cyst, mandible, cystic bone lesion, bone ostectomy

## Abstract

Mandible cemento-osseous dysplasia (COD) can be found mostly associated with dental roots and tooth-bearing anatomical structures. A variety of odontogenic cysts and tumors might have similar appearances. A lesion in the jaw bone not associated with dental roots with a cyst-like appearance might suggest a non-odontogenic lesion, an empty bone cavity, an osseous, fibrous, or fibro-osseous lesion, or a traumatic bone cyst (TBC). A radiolucent irregular bone cavity without clear borders always requires improved diagnostics in cone-beam computed tomography (CBCT) as well as a revision and a biopsy in some cases. When there is some bone swelling and asymmetry on radiological evaluation, followed by extra-cortical spread, and the lesion has irregular borders with thickening or atypical calcifications, a biopsy should be performed. COD and TBCs can be found mostly associated with dental roots, but sometimes they are not associated with tooth-bearing jaw structures and might cause some diagnostic problems, especially if they resemble an empty radiolucent cystic-like lesion in an atypical location. Regardless of the type of lesion, a bone revision and a biopsy are important. When a sufficient amount of a sample is removed and evaluated, this can greatly improve the final diagnosis. The authors present an interesting case of a lesion accidentally found in a routine panoramic radiograph used for screening before scheduled orthodontic treatment.

**Figure 1:**
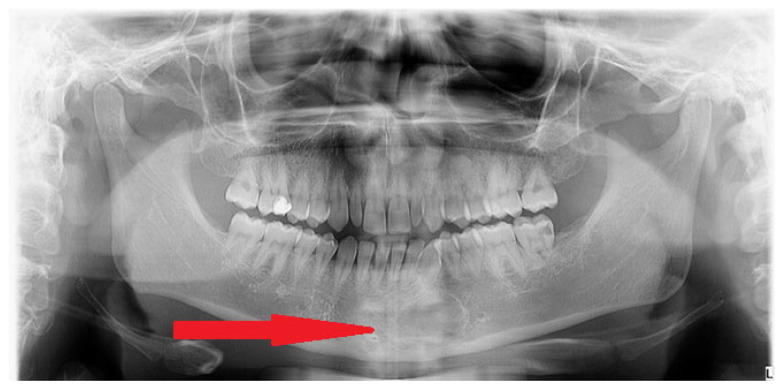
A 26-year-old generally healthy male was scheduled for orthodontic treatment. Before the treatment during a routine panoramic screening, an atypical radiolucent area was found in the anterior mandible (red arrow). The scope of many bone diseases can manifest as different radiolucent, radiopaque, or mixed radiolucent–radiopaque features. Bone changes of odontogenic origins are quite well known and established. Some special consideration should be focused on non-odontogenic jaw diseases of various types. The WHO (World Health Organization) recently distinguished fibrous, osseous, and fibro-osseous jaw bone lesions. Cemento-osseous dysplasia (COD) is one of the most common benign fibro-osseous jaw lesions and is characterized by different shapes, sizes, and maturation levels. Quite often, COD presents itself when mature bone is replaced with cementum or immature woven bone surrounded by moderate or low levels of fibrous connective stroma, commonly found in the tooth-bearing bone structures or outside of those areas. Typically, COD has four forms, named focal, periapical, florid, and familiar. Because of many clinical, radiologic, and histopathological similarities, a final description of any non-odontogenic bone lesion should include cemento-osseous dysplasia, ossifying fibroma (OsF), fibrous dysplasia (FD), and osteoblastoma (OSB), as all of them may exhibit some similar histologic, clinical, and radiographic characteristics. On the other hand, dense bone islands (DBIs, idiopathic bone osteosclerosis—IBO), condensing osteitis (CO), complex odontoma, periapical osteoma, ectopic calcification, exostoses (tori), hypercementosis, atypically dense alveolar bone, and other bone dysplasia have more radiopaque appearances [1,2]. Many of these mentioned lesions are found accidentally on routine panoramic radiographs before orthodontic treatment [3]. Mixed radiolucent and radiopaque areas with combined radiological appearance and heterogeneous structures of various shapes, borders, radiolucent rims, and calcifications within are very troublesome in diagnostics and management. Other pathologies and lesions found in the jaw can include the following differential diagnoses: Paget Disease, root remnants, cementoblastoma (CB), odontomas, diffuse sclerosing chronic osteomyelitis (Garre), and focal sclerosing osteomyelitis, and also Brown Tumors in primary hyperparathyroidism, multiple myelomas, osteoporotic bone marrow space, and periapical inflammation/granuloma formation [4]. In each lesion with irregular borders and unclear radiological and clinical appearance, a detailed evaluation should be performed first in a CBCT (cone-beam computed tomography study). It is worth noting that some benign bone lesions have various patterns of appearance based on their stage of growth, bone maturation, and changes like calcification, ossifications, or similar changes within their mass [2,3,4].

**Figure 2:**
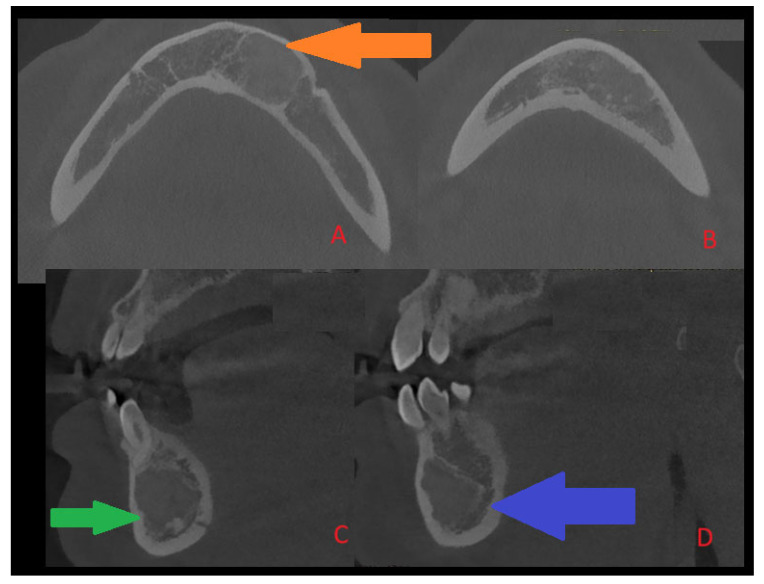
A typical COD is quite often a benign lesion, nonexpansile, asymptomatic, associated with vital teeth, found accidentally on radiographs, quite commonly near teeth apices or under/between them, and can rarely cause bone expansion, swelling, and pain associated with secondary inflammation or other tooth-related infection. The presence of any bone borders of COD and its rim and structure are greatly related to its radiolucent/radiopaque or mixed form of maturation and growth, as is the occurrence of an isolated form of any single periapical teeth lesion (near apices), and this can vary to forms of multifocal, multiquadrant, and advanced jaw lesions. In most cases, simple clinical and surgical observation is sufficient, along with teeth vitality testing. Rarely, in its maturation stage, its sclerotic, fused, or entirely lucent structures can mimic cysts, bone cavities, or other pathologies. Most commonly, teeth are non-displaced, and cortical expansion and swelling are not present. In the following case (presented CBCT scans: CBCT 20 × 20 cm FOV (field of view) imaging protocols based on RayScan S 5471.3 mGy (Ray Company Co., Ltd., Samsung 1-ro, Hwaseong-si, Gyeonggi-do, Republic of Korea)), CBCT evaluation was carried out in RAYSCAN S without slicing, with an average thickness between 0.070 mm and 0.3 mm. Exposure time was 16 s with an average voltage of 4–17 mA and current of 60–90 kV. Because of some bone swelling ((**A**), orange arrow), followed by irregular cystic lining (green arrow, (**C**)) and a less visible and unclear radiolucent and radiopaque ((**B**,**D**), blue arrow) appearance in the posterior and inferior parts of the lesion, the radiological image was unclear. Because of the following, in this case, suspicion of an expansile lesion was raised. Firstly, a type of cystic-like ameloblastoma (UA), traumatic bone cyst (TBC), arteriovenous malformation (AVM), or other uncertain odontogenic tumor-like lesion origin was suspected. In the following case, a typical small window biopsy was avoided. In order to obtain greater visibility of the lesion, evaluate the bone directly, and gather a good number of specimens for histopathological and microbiological examination, and if necessary, prepare for any serious bleeding, fracture, or bone mobility, a procedure under general anesthesia was scheduled [3,4].

**Figure 3:**
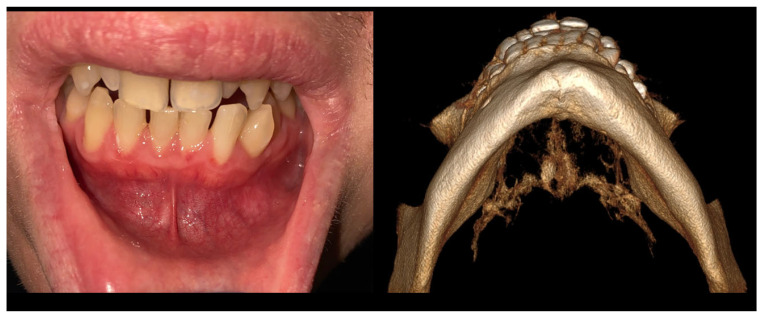
During extraoral examination, there were slight swellings and asymmetry at the lower border of the mandible. Intraoral examination revealed the absence of extensive swellings and bone crepitation or pathological mucosal signs. All teeth were normo-responsive at the vitality test. Orthopantomography (Figure 1) revealed the presence of an atypical radiolucent irregular area surrounded by a radiolucent/radiopaque border, without well-defined margins, at the radicular apices of teeth 32–34. In the comparison of bone swelling in clinical and radiologic images, some atypical, slight cortical bone asymmetry and elevation were noted. In the CBCT at the periapical area of the teeth, the lesion was not attached to the roots, and the neuro-vascular bundle from the teeth apex was clearly visible. The radiographic image of the lesion showed about 15 mm of major diameter without clear borders (Figure 2). Biopsy revealed a cyst-like cavity without any typical mass, solid structures, or fluid; just a few tissue remnants were found. After sample gathering, a curettage of the bone cavity was performed. After the biopsy, the healing was uneventful. Because of this lesion’s irregular appearance, lack of association with teeth roots, and atypical borders, a differential diagnosis of other odontogenic tumors, jaw bone lesions (fibrous, fibro-osseous, or osseous), or traumatic bone cysts or bone cyst-like malformations should be established. In order to exclude any odontogenic cyst or periapical teeth lesions, firstly, a good clinical patient chart anamnesis, followed by testing for teeth vitality on cold stimulus, might be helpful. Secondly, cortical bone expansion, the status of the dental root (displaced, resorption), the presence of a thin sclerosing border of the lesion, and various small, differently shaped and sized calcifications visible on any radiograph might help in diagnostics. Since some lesions of both odontogenic and non-odontogenic origin can look exactly the same, a revision surgery with biopsy should be used when radiological appearance and the patient’s surgical anamnesis are not sufficient for any final diagnosis, and when teeth are excluded as the potential source of the lesion. Other worrisome symptoms might include extra-cortical spread, loss of sensation (Vincent sign), teeth resorption, or even a pathological fracture. On the other hand, cystic-like lesions with clear borders, without any calcification within, and with non-displaced or resorbed teeth might indicate the presence of a TBC. Both a TBC and COD would require a revision and curettage to check the cavity borders and structure, check for the presence of any fluid or solid mass, and also promote bone healing because of cavity scarification and blood clot formation [4,5,6,7].

**Figure 4:**
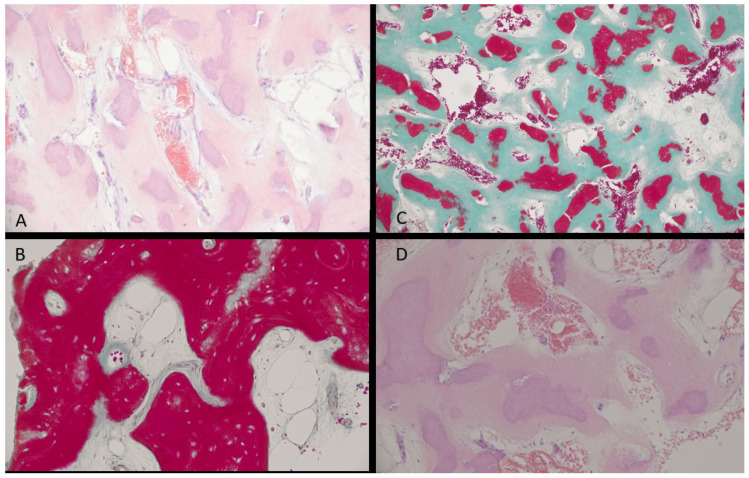
Only a good histopathological examination could fully estimate the scope of any irregular jaw bone lesion. Because some bone cystic-like cavities can look quite similar in radiological evaluation, either improved diagnostics with CBCT or a revision surgery for biopsy of the lesion might influence the final diagnosis. The material is not separated from the other tissues by a capsule; therefore, the lesion blends into the surrounding bone and other tissues. This is found in Figure 1 and Figure 2. A detailed description includes the following: (**A**)—Loose connective tissue adjacent to the lesion. The stroma of the sample is composed of spindle-shaped fibroblasts and collagen fibers with numerous large blood vessels (capillaries). (**B**)—Stroma of the lesion with cystic changes. In the stroma, islets of woven and lamellar bone are highly mineralized. Moreover, cementum-like calcifications (rounded, basophilic droplets) can be spotted in H&E staining. (**C**)—Note the cementum-like calcifications in bone. No osteoid deposits were noted within the material. The material was highly vascular with a lower number of osteoblasts and other cells. This is typical for a more advanced stage of cemento-osseous dysplasia (COD). (**D**)—Locally, microcystic changes are visible between bone trabeculae. Note the cystic features in the specimen. Radiologically, an irregular cystic-like cavity without any sign of radiopaque masses or calcification within the lesion is hard to evaluate. While a TBC is an empty bone cavity with a cyst-like lesion appearance, it does not have any lining itself. The COD, on the other hand, and its radiological appearance might be misjudged or misinterpreted; therefore, in histopathological examination, a calcified material with numerous smaller calcified-like masses with droplets of cementum-like material resembling a ginger root-like formation is quite common; however, the COD stage of growth and maturation might be different in each case. Because of the following differences in each stage of COD growth, this lesion can mimic other bone lesions, bone cavities, cysts, and tumors of different origins. In cases of both COD and TBCs, when the cyst-like cavity is mostly free of any cyst lining or lesion/tumor mass, it is crucial to perform an extensive curettage to gather some parts of the material for examination [8].

**Figure 5:**
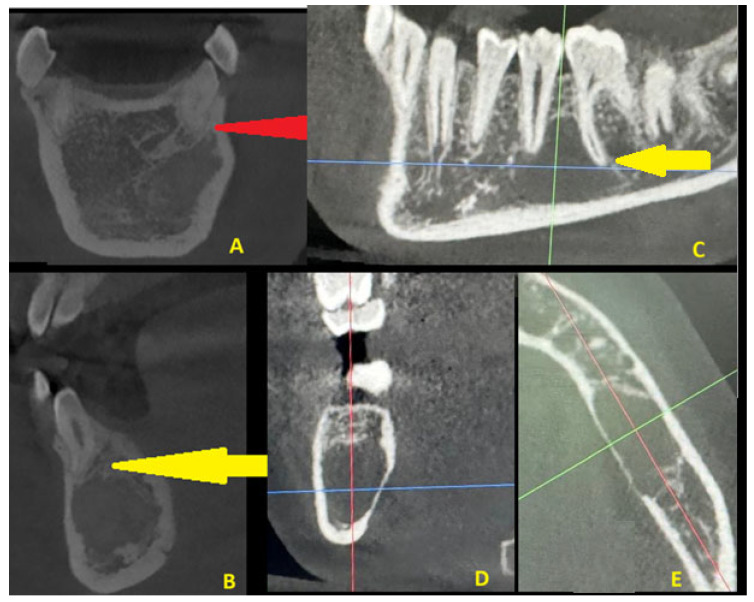
A TBC is an unusual, non-neoplastic, accidentally found pseudocystic cavity, common in the posterior mandible, which is asymptomatic, without any clinical relevance, and slow-growing (**C**–**E**). It is mostly found in children and young adults, and sometimes its occurrence might be related to minor trauma. In both cases, trauma or other injuries to the mandible were not confirmed. The lesion itself is an empty bone cavity, without any lining, without calcifications, and it commonly surrounds teeth roots or embeds them within the lesion (**C**–**E**). Clinically, teeth remain vital, and the neuro-vascular bundle is intact (yellow arrow) in both COD and TBCs. Normally, COD lesions (**A**,**B**) are associated with teeth roots or apices, similar to a TBC; however, sometimes they can be found far from any tooth-bearing structures, which might cause misdiagnosis (red arrow). A radiological comparison in CBCT between COD and a TBC is presented in Figure 5. In a TBC, a radiolucent unilocular lesion has mostly scalloped margins and is either empty or filled with blood or some atypical fluid-like substance. Because both lesions can look like cystic-like lesions, and some lesions of both odontogenic and non-odontogenic origin can look exactly the same, revision surgery with biopsy should be used when radiological appearance and the patient’s surgical anamnesis are not sufficient for any final diagnosis [9,10]. Sometimes, a comparison with future radiographs and a wait-and-see approach is a solution, but each case requires some individual treatment. A study by Park et al. highlights that CBCT is the most accurate diagnostic tool to distinguish COD from other pathologies found in the tooth-bearing regions of the jaws, especially other cyst lesions [5]. On the other hand, additional diagnostics like SPECT (single-photon emission tomography) with blood evaluation for calcium–phosphorus markers are also needed in some cases of endocrine-related jaw bone lesions [1,2,3,4,5,6]. The red, blue, and green axes (**C**–**E**) represent the field of radiological visualization of the TBC. To summarize, improved diagnostics with CBCT is quite important to underline the scope of the lesion and the possible necessity for a biopsy. Secondly, any lesions without any calcifications, septa formation, or bone structures within the cystic cavity should be differentiated between a unicystic ameloblastoma, a traumatic bone cyst, early stages of cemento-osseous dysplasia formation, or similar cystic lesions, as all of the mentioned conditions look very similar in CBCT. When a similar radiological appearance is found, revision surgery followed by a biopsy is the most advised diagnostic step to minimize any risk of false diagnosis.

## Data Availability

The datasets used and/or analyzed during the current study are available from the corresponding author upon reasonable request.

## References

[1] Mishra G., Bernisha R., Bhogte S.A., Chitra P. (2024). Idiopathic Osteosclerosis in Orthodontic Patients: A Report of Two Cases. Cureus.

[2] Nelke K., Matys J., Janeczek M., Małyszek A., Łuczak K., Łukaszewski M., Frydrych M., Kulus M., Dąbrowski P., Nienartowicz J. (2024). The Occurrence and Outcomes of Cemento-Osseous Dysplasias (COD) in the Jaw Bones of the Population of Lower Silesia, Poland. J. Clin. Med..

[3] Smołka P., Nelke K., Struzik N., Wiśniewska K., Kiryk S., Kensy J., Dobrzyński W., Kiryk J., Matys J., Dobrzyński M. (2024). Discrepancies in Cephalometric Analysis Results between Orthodontists and Radiologists and Artificial Intelligence: A Systematic Review. Appl. Sci..

[4] Noffke C.E.E., Raubenheimer E.J., Peranovic V. (2019). Cemento-osseous dysplasia: A diagnostic challenge. S. Afr. Dent. J..

[5] Park S., Jeon S.J., Yeom H.G., Seo M.S. (2024). Differential diagnosis of cemento-osseous dysplasia and periapical cyst using texture analysis of CBCT. BMC Oral Health.

[6] Lis E., Gontarz M., Marecik T., Wyszyńska-Pawelec G., Bargiel J. (2024). Residual Cyst Mimicking an Aggressive Neoplasm—A Life-Threatening Condition. Oral.

[7] Gumru B., Akkitap M.P., Deveci S., Idman E. (2021). A retrospective cone beam computed tomography analysis of cemento-osseous dysplasia. J. Dent. Sci..

[8] Urs A.B., Augustine J., Gupta S. (2020). Cemento-osseous dysplasia: Clinicopathological spectrum of 10 cases analyzed in a tertiary dental institute. J. Oral Maxillofac. Pathol..

[9] Decolibus K., Shahrabi-Farahani S., Brar A., Rasner S.D., Aguirre S.E., Owosho A.A. (2023). Cemento-Osseous Dysplasia of the Jaw: Demographic and Clinical Analysis of 191 New Cases. Dent. J..

[10] Brody A., Zalatnai A., Csomo K., Belik A., Dobo-Nagy C. (2019). Difficulties in the diagnosis of periapical translucencies and in the classification of cemento-osseous dysplasia. BMC Oral Health.

